# Differential Preclinical Efficacy of Combined CDK4/6 and MEK Inhibition in Low-Grade Serous Ovarian Carcinoma Based on *KRAS/NF1* Mutational Status

**DOI:** 10.3390/ijms27041774

**Published:** 2026-02-12

**Authors:** Madison Bittner, Marta Llaurado Fernandez, Joshua Hoenisch, Yuen Yee Leung, Hannah Kim, Nelson K. Y. Wong, Kathleen I. Pishas, Dane Cheasley, Karla J. Cowley, Kaylene J. Simpson, Yen-Yi Lin, Stanislav Volik, Stephane Le Bihan, Colin C. Collins, Martin Köbel, Mark S. Carey

**Affiliations:** 1 Department of Obstetrics and Gynecology, University of British Columbia, Vancouver, BC V6H 3N1, Canada; 2 Department of Experimental Therapeutics, BC Cancer, Vancouver, BC V5Z 1L3, Canada; 3 Peter MacCallum Cancer Centre, Melbourne, VIC 3000, Australia; 4 Sir Peter MacCallum Department of Oncology, The University of Melbourne, Melbourne, VIC 3010, Australia; 5 Victorian Centre for Functional Genomics, Peter MacCallum Cancer Center, Melbourne, VIC 3000, Australia; 6 Vancouver Prostate Centre, Vancouver, BC V6H 3Z6, Canada; 7 Department of Urologic Sciences, University of British Columbia, Vancouver, BC V5Z 1M9, Canada; 8 Department of Pathology and Laboratory Medicine, University of Calgary, Calgary, AB T2N 1N4, Canada; 9 Department of Clinical Research, BC Cancer, Vancouver, BC V5Z 1L3, Canada

**Keywords:** ovarian cancer, low-grade serous ovarian carcinoma, MAPK, p16/*CDKN2A*, targeted therapies, palbociclib, trametinib

## Abstract

Low-grade serous ovarian carcinoma (LGSOC) usually presents in advanced stages and is associated with a high mortality rate. Clinical trials targeting the MAPK and cell cycle pathways in LGSOC have shown promising results for its treatment, however there is a need to improve efficacy and define predictive biomarkers to guide patient selection for treatment using these agents. We therefore evaluated cell cycle protein expression by immunohistochemistry (IHC) in 186 LGSOC cases, and evaluated the efficacy of the MEK inhibitor, trametinib, in combination with the CDK4/6 inhibitor, palbociclib, in preclinical models of LGSOC. Abnormal p16 expression was observed in 20% of primary and 46% of recurrent tumors, and it was associated with poorer survival (log-rank *p* = 0.005). Notably, cell lines with increased sensitivity to trametinib were more likely to harbor mutations in *KRAS* or *NF1* and displayed low pRb levels. Palbociclib showed limited efficacy in vitro; however, the combination of palbociclib and trametinib treatment produced synergistic antiproliferative effects in *KRAS/NF1*-wild-type cell lines, which displayed higher pRb levels. Acquired drug resistance was linked to increased cyclin D1/E1 expression. This study confirms abnormal p16 IHC as a negative prognostic marker in LGSOC and establishes key determinants of sensitivity to CDK4/6 inhibitor-based therapy.

## 1. Introduction

Low-grade serous ovarian carcinoma (LGSOC) is recognized as a distinct entity for its unique clinical, pathological, and molecular features [1]. As a rare cancer it accounts for only 2–5% of all ovarian carcinomas and approximately 1500 cases are diagnosed annually in Canada and the United States [1,2]. Compared with high-grade serous ovarian cancer (HGSOC), LGSOC occurs in younger women (median age 45–55 vs. 63 years) and exhibits relative chemoresistance [2,3,4,5]. Standard treatment usually consists of cytoreductive surgery then platinum-based chemotherapy followed by hormonal maintenance therapy [5,6]. As most LGSOC present in advanced stages (stage III–IV), and current therapies are not curative, fewer than 50% of patients survive more than 10 years [5,7,8].

Molecular profiling has identified a small set of recurrent genetic alterations that can be classified in the following categories: (1) mitogen-activated protein kinase (MAPK) pathway mutant or aberrant (~30%); (2) EGFR high (~25%); (3) FOLR1 high (~25%); (4) progesterone receptor negative, with high fraction of genomic alterations (~20%); (5) *CDKN2A*/p16 abnormal (~15%, either absent or block type); and (6) *USP9X* mutant (~10%) [9,10,11,12,13,14,15]. These subtypes, while not mutually exclusive, provide key prognostic and therapeutic insights.

Emerging therapies against LGSOC are mostly focused on targeting two signaling pathways: the MAPK and the cell cycle pathway. Randomized clinical trials using MEK inhibitors (MEKi) have resulted in the approval of trametinib (TRA) for the treatment of relapsed disease [10,16,17,18]. However, it is still unclear whether mutations in *RAS/RAF* are predictive biomarkers. In GOG-281, despite improvements in response rates (RR) to TRA in *KRAS/BRAF/NRAS*-mutant versus wild-type tumors (RR 50% vs. 8.3%, respectively) and improved progression-free survival (PFS) (13.0 vs. 7.2 months), these differences were not statistically significant [10]. Although MEK inhibition has improved outcomes for patients with relapsed LGSOC, the overall RR is only 26% and considering its toxicities, the therapeutic benefit in some patients is limited [14]. MEKi-based combination strategies are being evaluated to enhance efficacy including those with cyclin-dependent kinase 4/6 inhibitors (CDK4/6i), anti-hormone therapy (AHT), focal adhesion kinase inhibitors (FAKi), and BCL-2 inhibitors (BCL2i) [15,16,19,20,21,22]. Furthermore, recognizing the low RR to AHT in relapsed LGSOC (which is estimated to be around 9%), CDK4/6i were evaluated to overcome endocrine resistance as in breast cancer [23,24,25]. Early-phase trials using CDK4/6i and AHT combination in LGSOC have shown encouraging activity, with response rates ranging from 47% in advanced-stage to 23% in recurrent settings [23,26]. To properly identify those patients who are likely to benefit from CDK4/6i therapy predictive biomarkers are needed.

MEKi and CDK4/6i combinations emerged as a promising strategy to suppress sustained cell cycle activation observed in *KRAS*-mutant cancers such as breast, colon, and pancreatic malignancies [27,28,29]. Preclinical studies in these cancers demonstrated synergistic efficacy of MEKi and CDK4/6i, prompting a phase II clinical trial of this combination in recurrent LGSOC [30]. However, the efficacy of these agents, alone or in combination, in the context of the molecular alterations seen in LGSOC is unknown. Thus, in this present study we sought to describe the expression of key cell cycle markers in LGSOC tumors and evaluate the preclinical efficacy of CDK4/6i (palbociclib, PLB) and MEKi (TRA) therapies in LGSOC models with defined *CDKN2A*/p16 and MAPK profiles.

## 2. Results

### 2.1. Loss of p16 Expression Is Common in Recurrent LGSOC Tumors and Correlates with Worse Patient Survival Outcomes

*CDKN2A* (p16) is, after TP53, the second most commonly inactivated tumor suppressor gene in cancer [31]. It acts as a natural inhibitor of CDK4/6 to stop cell cycle progression. CDK4/6i were developed to selectively block CDK4/6 activity, targeting cancers driven by aberrant CDK4/6-cyclin D1 signaling [32]. Thus, LGSOCs lacking *CDKN2A*/p16 are predicted to be more sensitive to these therapies [33].

To better understand the frequency of p16 alterations in LGSOC tumors and help guide the future use of CDK4/6i therapies in this disease, we first evaluated the expression of a set of key cell cycle protein regulators in primary-advanced and recurrent tumors. Immunohistochemical (IHC) staining for p16 was performed on five independent tissue microarrays (TMAs) containing 186 LGSOC cases [13]. Among these, 155 cases consisted of primary-advanced (stage III–IV) tumors, which represent those patients in urgent need of effective systemic treatments. Interpretable p16 IHC data was obtained from 146 cases. Our results showed that while most primary-advanced LGSOC tumors displayed normal p16 expression (80.1%), abnormal p16 expression was detected in about 1/5 of cases (19.9%) (Table 1, Figure S1). In particular, the absence of p16 expression was observed in 15.8% of cases and block p16 expression in 4.1% of cases. Core tissue staining for p16 was validated in a subset of LGSOC cases using full tumor sections and a concordance rate of 100% was observed (Table S1).

To further investigate the cell cycle profiles of LGSOC tumors, we evaluated cyclin D1, cyclin E1 and retinoblastoma (Rb) IHC expression in a subset of cases. While most tumors expressed low levels of cyclin E1 expression (98.9%), close to half of them (49.4%) expressed moderate to high levels of cyclin D1 expression (27.6% and 21.8%, respectively). All cases were found to retain total Rb expression (Table 1). Furthermore, no correlation between p16 and cyclin D1 expression was detected (*p* = 0.775) (Table S2).

Next, we compared p16 IHC expression between primary and recurrent LGSOC tumors. To do so, we obtained tissue sections from 26 independent recurrent tumors that matched 26 primary tumors included in our TMAs. Among these cases, abnormal (absence or block) p16 expression was detected in 15.4% of primary and 46.2% of recurrent tumors, representing a three-fold increase in its frequency (Table 2). In particular, the absence of p16 expression was observed in 11.5% of primary tumors (all stages) and 38.5% of recurrent tumors, and block p16 expression was observed in 3.8% of primaries and 7.7% of recurrent tumors. Over time, acquisition of p16 abnormality was detected in over 30% (8/26) of cases, with seven cases losing p16 expression and one case acquiring block p16 expression at recurrence.

**Figure 1:**
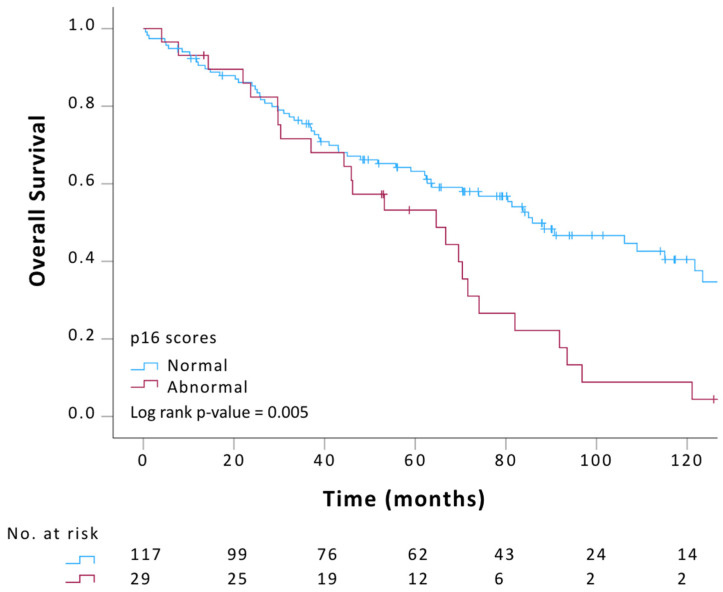
Kaplan–Meier overall survival curve in primary-advanced (III–IV) LGSOC patients (n = 146) with distinct p16 immunohistochemistry tumor expression. Univariable survival analysis, *p* = 0.005.

Lastly, we interrogated the potential correlation between p16 tumor expression and other clinical variables in primary-advanced LGSOC patients. Clinical variables included: age, tumor type (primary vs. recurrent), stage, residual disease, tumor of origin (ovarian vs. peritoneal), treatment type and overall survival (OS) outcome. The median follow-up of the patient population was 83.8 months. The results from our statistical analysis demonstrated a significant correlation between abnormal p16 tumor expression and recurrent disease (Pearson Chi-Square *p* < 0.001, Table 3). Additionally, the results of our univariable Kaplan–Meier survival analysis showed that among primary-advanced (stage III–IV) LGSOC patients, in which survival is already known to be poor, those with abnormal p16 tumor expression were associated with a worse survival outcome than those with normal p16 tumor expression (Figure 1; log-rank, Mantel–Cox, *p* = 0.005). The curve highlights a late separation with a 10-year survival of about 40% for p16 normal primary LGSOC versus 10% for p16 abnormal cases. Finally, the results from our multivariable analysis indicated that both residual disease and p16 expression were statistically significant and thus likely independently contributing to the survival outcome (Cox-regression, *p* = <0.001 and *p* = 0.043, respectively). Details of the multivariable analysis are included in the Supplementary Table S3, and the Kaplan–Meier survival curves comparing normal versus abnormal–absent and abnormal–block are included in the Supplementary Figure S2.

### 2.2. LGSOC Cell Lines Often Lack p16 Expression, Representing a More Aggressive Subtype of the Disease

**Figure 2:**
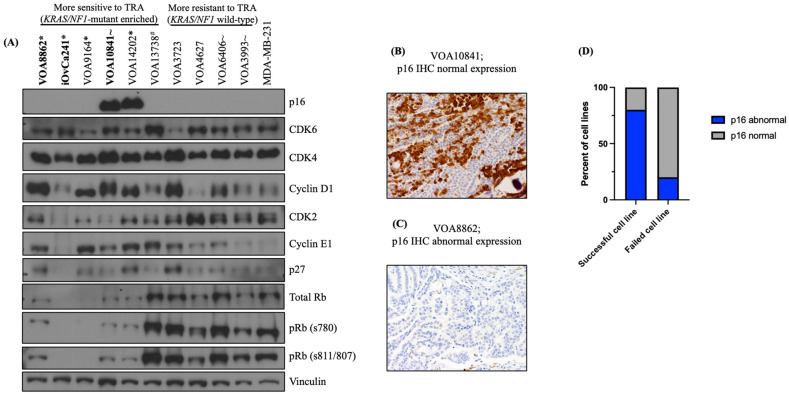
Cell cycle protein expression in LGSOC tumors and cell lines. (**A**) Expression of cell cycle markers in 10 LGSOC cell lines by Western blot. TRA sensitivity classification was based on cell proliferation data and resistant biomarker profiles (PKCa and EGFR), as previously published [34,36]. The MDA-MB-231 breast cancer cell line was used as a protein loading control. Confirmed pathogenic mutations noted: (*) *KRAS*, (#) *NF1* and (~) *NRAS*. (**B**) LGSOC tumor with normal p16 immunohistochemistry (IHC) expression and (**C**) LGSOC tumor without p16 IHC expression. (**D**) Frequency of p16 expression in successful and failed cell lines derived from LGSOC tumors.

In our previous work, we showed that most LGSOC cell line models (86%; 12/14) lack p16 expression and that *CDKN2A*/p16 loss may be a putative new molecular driver of LGSOC disease [34]. In the present study, we sought to investigate whether the loss of p16 expression observed in the successfully established models (Figure 2D) resulted from experimental biases stemming from patient selection, tumor type and/or cell culture conditions. To do so, we screened 10 LGSOC cell lines representing 10 independent LGSOC patients that were established and characterized (at pathological, clinical and molecular level) in our laboratory [34,35,36]. Among these, four (4/10, 40%) were derived from primary and six (6/10, 60%) derived from recurrent LGSOC tumor samples (Table S4). Only two (2/10, 20%) cell lines retained p16 expression by Western blot (WB) (Figure 2A): one was derived from a primary-advanced tumor with normal p16 expression (VOA10841) (Figure 2B) and the other was derived from a recurrent tumor with abnormal–block p16 expression (VOA14202) (Table S4). Of note, both of these lines were derived from tumors carrying MAPK mutations (VOA10841, *NRAS*-mutant; VOA14202, *KRAS*-mutant). In contrast, the remaining eight (8/10, 80%) cell lines lacked p16 expression by WB (Figure 2A) and, among those with banked tumor for IHC testing, 80% (4/5) of the corresponding tumors were p16-negative (Figure 2C,D; Table S4). Hence, among the seven cases with matching tumor tissue, we observed concordant p16 expression between LGSOC cell lines and tumors in 85% (6/7) of cases. Thus, only one case (VOA13738) showed discrepant p16 IHC-WB results, normal p16 expression in the tumor tissue and an absence of p16 expression in the matching cell line. Overall, as WB cannot distinguish between normal and block p16 in cell lines, and taking the parental tumor IHC result into consideration, 9/10 established LGSOC cell lines have an abnormal p16 status.

Next, we aimed to evaluate p16 IHC expression in 10 independent LGSOC tumors, from which our cell line development attempts had failed. Most of these cases were primary-advanced tumors and, contrary to what we observed in the tumors with successfully established cell lines, eight of them (8/10, 80%) displayed normal p16 expression and only two (20%) displayed abnormal (absent or block) p16 expression (Figure 2D, Table S4).

### 2.3. KRAS/NF1-Mutant LGSOC Cell Lines Do Not Respond to Dual MEK and CDK4/6 Inhibition and Have Low Levels of Rb Expression

Using our collection of patient-derived LGSOC cell line models, we evaluated CDK4/6i alone and in combination with MEKi. To assess the most potent CDK4/6 inhibitor, 12 LGSOC cell lines were screened against 18 inhibitors of CDK4/6; including two LGSOC cell line models established in Australia (AOCS2 and SLC58) [37]. As a toxicity control, all compounds were screened against a cell line derived from normal ovary surface epithelium (IOSE-523). In total 55.5% (10/18) of compounds demonstrated no cytotoxicity in IOSE-523 cells (robust Z-score > −1.0) at the lowest dose assessed (0.1 μM). Using a robust Z-score cutoff of <−2 to indicate cytotoxicity, PLB was the most potent (8/12 cell lines) across the LGSOC cell line panel (mean robust Z-score −2.241 ± 0.235) with no toxicity in IOSE-523 cells observed (robust Z-score 0.355). The next most potent was abemaciclib (7/12 cell lines, mean robust Z-score −1.933 ± 0.318). Although ribociclib exhibited no cytotoxicity in IOSE-523 cells (robust Z-score 1.011), cytotoxicity was not observed in any LGSOC cell line (mean robust Z-score 0.066 ± 0.206) (Figure S3).

**Figure 3:**
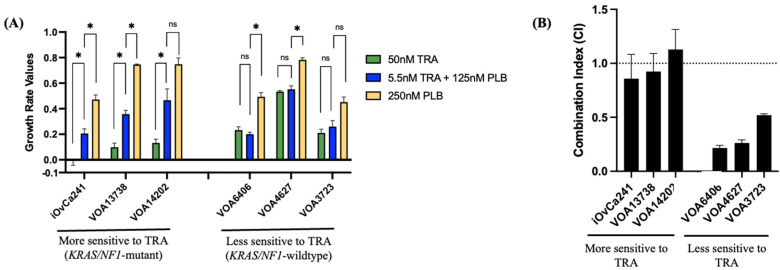
Preclinical efficacy of TRA and PLB treatment in LGSOC cell lines. (**A**) Growth rate (GR) values of 90 h high-dose TRA (50 nM), high-dose PLB (250 nM) and low-dose TRA plus PLB combination (TRA 5.5 nM + PLB 125 nM) in LGSOC cell lines with distinct *KRAS/NF1* gene status. (**B**) Combination index (CI) of 90 h low-dose TRA and PLB combination (TRA 5.5 nM + PLB 125 nM) in LGSOC cell lines with distinct *KRAS/NF1* gene status. Note: Cell line VOA14202 showed p16 expression by Western blot; later confirmed to be derived from a tumor with block–abnormal p16 expression. (*) *p*-value less than 0.05. (ns) Not significant.

Next, we selected 10 LGSOC cell lines for further evaluation of PLB and TRA. These included four derived from primary-advanced LGSOC tumors and six derived from recurrent LGSOC tumors; all representing LGSOC patients in need of effective systemic treatment. Among these lines, seven have previously been tested for TRA sensitivity and three were newly derived and tested (Table S5; Figure S4) [34,35,36]. Our sequencing analyses revealed that four lines carried oncogenic *KRAS* mutations, one line carried a loss-of-function *NF1* mutation (which results in constitutive RAS activity [38]), three lines carried oncogenic *NRAS* mutations and two lines were *KRAS/NRAS/NF1*-wild-type (Table S5). In these lines, our preclinical evaluation of TRA treatment showed that TRA produced cell inhibitory effects in all LGSOC cell lines. However, as observed in our previous studies and recent MEKi trials in LGSOC patients, our new experimental data confirmed that those cell lines with oncogenic *KRAS/NF1* mutations (n = 5) displayed a higher degree of sensitivity to single TRA treatment compared to those with *KRAS/NF1*-wild status (n = 5) (50 nM TRA growth rate (GR) values ranging from −0.010 to 0.133 in *KRAS/NF1*-mutant lines vs. 0.210 to 0.534 in *KRAS/NF1*-wt lines) (Figure 3A; Table S6) [10,34,35,36,39]. Of note, among the three cell lines with oncogenic *NRAS* mutations, two cell lines carried a *NRAS* p.Q61R mutation and were found to be more resistant to high TRA treatment when compared to the cell line that carried a *NRAS* p.Q61K mutation (Figure 2). Interestingly, among all 10 LGSOC cell lines, those derived from primary-advanced tumors (4/4, 100%) displayed a higher degree of sensitivity to TRA than those derived from recurrent tumors (2/6, 33.3%) (Table S5).

To better understand CDK4/6i efficacy in relation to MEKi sensitivity, we selected three *KRAS/NF1*-mutant cell lines (one with abnormal–block p16 expression; VOA14202), and three *KRAS/NF1*-wild-type cell lines (all lacking p16 expression) (Figure 2A; Table S5). We selected drug doses that closely reflected the maximum human tolerated doses (250 nM for PLB and 50 nM for TRA) and reduced them accordingly (serial 1/2 doses for PLB and serial 1/3 doses for TRA, based on their known degree of cellular toxicity).

Our experimental results confirmed that single PLB treatment elicited low-to-moderate cellular toxicity across all LGSOC cell lines, with 250 nM PLB GR values ranging from 0.783 to 0.452 (Figure 3A; Table S6). The cell line with abnormal p16 block expression, VOA14202, was confirmed to be rather resistant to high-dose PLB treatment (250 nM PLB GR value = 0.748), with a similar degree of PLB resistance as two other p16-negative (absent) cell lines (250 nM PLB GR values: VOA4627 = 0.783; VOA13738 = 0.745). Interestingly, while all LGSOC cell lines expressed abnormal p16 expression, only 50% of them displayed moderate sensitivity to high-dose (250 nM) PLB treatment. Specifically, two out of three *KRAS/NF1*-wt cell lines (250 nM PLB GR values: VOA3723 = 0.452; VOA6406 = 0.493) and one out of three *KRAS/NF1*-mutant cell lines (250 nM PLB GR value: iOvCa241 = 0.472) (Table S6).

Testing PLB and TRA in combination elicited differential cellular responses between *KRAS/NF1*-wild-type and *KRAS/NF1*-mutant LGSOC cell lines (Figure 3B and Figure S5; Table S6). In particular, PLB and TRA combination produced synergistic drug effects (combination index, CI < 1) in the *KRAS/NF1*-wild-type cell lines but not in the *KRAS/NF1*-mutant cell lines (Figure 3B and Figure S5A). Thus, low-dose TRA and PLB combination (5.5 nM TRA + 125 nM PLB) achieved similar cell inhibitory effects as high-dose TRA treatment (50 nM) in the *KRAS/NF1*-wild-type cell lines only. On the contrary, low-dose combination elicited antagonistic drug effects (CI > 1) and inferior cell inhibitory effects compared to high-dose TRA treatment (50 nM) in the *KRAS/NF1*-mutant lines (Figure 3A,B; Table S6). These findings were supported by both drug synergy and cell GR calculations, and they did not seem to depend on the drug doses tested.

Finally, in order to better understand the differential effects of PLB and TRA observed between the *KRAS/NF1*-mutant and wild-type LGSOC cell lines, we evaluated the levels of expression of a selection of well-known cell cycle pathway regulators by WB. These included CDK2, CDK4, CDK6, cyclin D1, cyclin E1, p27 and total and phosphorylated Rb. Our results showed that LGSOC cell lines with reduced sensitivity to TRA (*KRAS/NF1*-wild-type cell lines) exhibited higher levels of Rb (total and phosphorylated) and lower cyclin E1 expression than the rest of the cell lines. In contrast, those lines with a higher sensitivity to TRA treatment (*KRAS/NF1*-mutant cell lines) were more likely to display lower levels of Rb (total and phosphorylated) and higher levels of cyclin E1 protein expression (Figure 2A). Of importance, while these observations require validation in additional LGSOC research models and/or tumor specimens, no other associations between the degree of TRA efficacy and the expression of cell cycle markers (p16, CDK4, CDK6, cyclin D1 or p27) were detected (Figure 2A).

### 2.4. Acquired Resistance to TRA Increases Resistance to PLB and Vice Versa: Resistance Mechanisms May Differ Between KRAS-Mutant and KRAS-Wild-Type LGSOC Cell Lines

Targeted therapies such as MEK and CDK4/6 inhibitors, though initially effective in mutation-driven cancers, often face acquired resistance. To investigate this process in LGSOC, we developed in vitro models of TRA and PLB resistance and assessed effects on signaling and drug response. Here, we selected two p16-negative (absent) LGSOC cell lines with different MAPK mutation profiles and degrees of response to TRA treatment: iOvCa241, a *KRAS*-mutant (G12D) line that was highly sensitive to TRA (50 nM TRA GR value = −0.010), and VOA6406, a *NRAS*-mutant (Q61R) line that displayed some resistance to TRA (50 nM TRA GR value = 0.232) (Table S6). Of note, the patient associated with the VOA6404 cell line was diagnosed with recurrent LGSOC disease and received MEKi (binimetinib) treatment after failing initial chemotherapy. While this patient showed initial response to MEKi based on CT scans and symptoms, patient tumor response did not meet the RECIST 1.1 criteria (Response Evaluation Criteria in Solid Tumors) and consequently treatment ended after 7 months.

**Figure 4:**
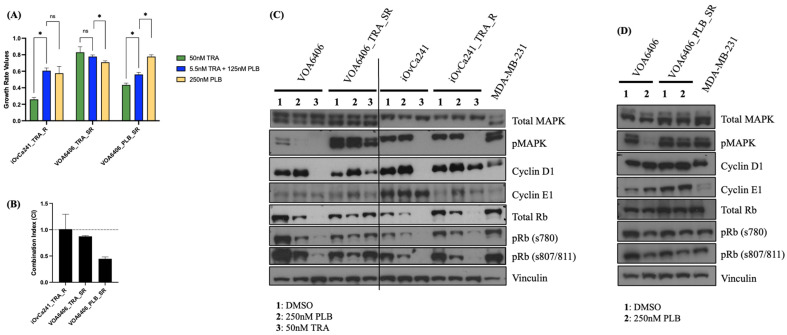
Effects of TRA and PLB treatment in LGSOC cell lines with acquired drug resistance. (**A**) Growth rate values of 90 h high-dose TRA (50 nM), high-dose PLB (250 nM) and low-dose TRA plus PLB combination (TRA 5.5 nM + PLB 125 nM) in TRA- and PLB-acquired drug-resistant LGSOC cell lines. (**B**) Combination index (CI) of 90 h low-dose TRA and PLB combination (TRA 5.5 nM + PLB 125 nM) in TRA- and PLB-acquired drug-resistant LGSOC cell lines. (**C**) Cell signaling effects of 48 h PLB (250 nM) and TRA (50 nM) treatments in two acquired TRA-resistant LGSOC cell lines compared to their corresponding parental cell lines by Western blot (WB). (**D**) Cell signaling effects of 48 h high-dose PLB (250 nM) treatment in an acquired PLB-resistant LGSOC cell line compared to its corresponding parental cell line by WB. The MDA-MB-231 breast cancer cell line was included as control in all WBs. (*) *p*-value of less than 0.05 was considered statistically significant.

Using these two cell lines, we developed two acquired TRA-resistant cell lines: the iOvCa241_TRA-R (TRA-resistant) cell line and the VOA6406_TRA_SR (TRA-super resistant) cell line. Both acquired TRA-resistant cell lines exhibited significantly increased GR in response to high-dose TRA treatment when compared to their corresponding parental cell lines (50 nM TRA GR values: 0.259 vs. −0.010 in the iOvCa241_TRA_R/iOvCa241 cell line pair, *p* < 0.05; and 0.830 vs. 0.232 in the VOA6406_TRA_SR/VOA6406 cell line pair, *p* < 0.05) (Figure 4 and Figure S6; Table S6). However, the degree of resistance to TRA treatment was noted to be superior in the VOA6406_TRA_SR cell line when compared to the iOvCa241_TRA_R cell line (50 nM TRA GR values: VOA6406_TRA_SR = 0.830; iOvCa241_TRA_R = 0.259) (Figure 4A; Table S6). Interestingly, this cell line also displayed increased resistance to PLB treatment when compared to its parental cell line (250 nM PLB GR values: 0.709 vs. 0.493 in the VOA6406_TRA_SR/VOA6406 cell line pair; *p* < 0.05). Finally, both acquired TRA-resistant cell lines experienced no or very limited synergistic drug effects in response to TRA and PLB combination (Figure 4B and Figure S5B). However, while high-dose TRA treatment was more efficacious than low-dose PLB and TRA combination in the iOvCa241_T_R cell line (GR values 50 nM TRA 0.259 vs. 5.5 nM TRA + 125 nM PLB 0.606, *p* < 0.05), low-dose PLB and TRA combination produced a similar inhibitory effect to high-dose TRA in the VOA6406_T_SR cell line (GR values 50 nM TRA 0.830 vs. 5.5 nM TRA + 125 nM PLB 0.777, *p* > 0.05). (Figure 4A; Table S6).

Both parental and acquired TRA-resistant cell line pairs showed notable differences in protein expression before and after treatment (Figure 4C). At basal level, the iOvCa241 cell line (*KRAS*-mutant/TRA-more sensitive) displayed higher levels of pMAPK (ERK1/2), lower levels of Rb (total and phosphorylated), and higher levels of cyclin E1 expression than the VOA6406 cell line (*KRAS*-wild-type, *NRAS*-mutant/TRA-less sensitive). After high-dose TRA (50 nM, 48 h) treatment, complete blockage of pMAPK and cyclin D1 levels were noted in both cell lines (iOvCa241 and VOA6404); however, full inhibition of pRb was only detected in the iOvCa241 cell line. These findings support the superior TRA sensitivity found in the iOvCa241 cell line when compared to the VOA6406 cell line (50 nM TRA GR values (iOvCa241 = −0.010 vs. VOA6404 = 0.232) (Figure 3A; Table S6). High-dose PLB (250 nM, 48 h) increased cyclin D1 and reduced Rb phosphorylation in both parental cell lines (iOvCa241 and VOA6406). However, a reduction in pMAPK expression levels was only detected in the VOA6406 cell line. Both cell lines displayed a similar degree of sensitivity to single PLB treatment (250 nM PLB GR values: iOvCa241 = 0.472 vs. VOA6406 = 0.493); however, the impact of PLB in the levels of pMAPK phosphorylation observed in the VOA6404 may help explain why this line displayed synergistic effects in response to PLB and TRA combination (Figure 3B; Table S6).

In the acquired TRA-resistant cell lines, cell signaling changes were also noted (Figure 4C). The iOvCa241_TRA_R cell line exhibited increased Rb (total and phosphorylated) and decreased cyclin E1 levels when compared to its parental cell line (iOvCa241). The VOA6406_TRA_SR cell line exhibited increased pMAPK (pERK1/2) and decreased Rb (total and phosphorylated) and cyclin D1 levels in comparison to its parental VOA6404 cell line (*NRAS*-mutant/TRA-resistant). High-dose TRA (50 nM, 48 h) treatment was no longer able to block but only reduce cyclin D1 expression levels in both acquired TRA-resistant cell lines. In addition, high-dose TRA blocked pMAPK and reduced pRb levels in the OvCa241_TRA_R line, but it only reduced pMAPK levels in the VOA6406_TRA_SR line. High-dose PLB (250 nM, 48 h) treatment, as seen in the parental cell lines, increased cyclin D1 and reduced total and pRb levels in both acquired TRA-resistant cell lines. However, while this treatment increased cyclin E1 expression in the iOvCa241_TRA_R cell line, this no longer reduced pMAPK levels in the VOA6406_TRA_SR cell line. Given that this cell line also spontaneously acquired additional resistance to PLB treatment (250 nM PLB GR values: VOA6406_TRA_SR = 0.709 vs. VOA6406 = 0.493, *p* < 0.05) (Figure S6; Table S6), it is possible that the inability of PLB to downregulate pMAPK levels may have contributed to this observation.

Lastly, we developed an acquired PLB-resistant cell line using the VOA6406 (*NRAS*-mutant) cell line, which had shown moderate response to PLB treatment and reduced sensitivity to TRA (standard-of-care) when compared to the iOvCa241 (*KRAS*-mutant) cell line. The resulting cell line, VOA6406-PLB-SR (super resistant), exhibited significantly increased GR in response to high-dose PLB treatment when compared to its parental cell line (250 nM PLB GR values: 0.778 vs. 0.493, *p* < 0.05) (Figure 4A; Table S6). Additionally, as previously observed in the VOA6406 cell line in which the acquisition of TRA resistance resulted in increased PLB resistance, in this line, the acquisition of PLB resistance also produced increased TRA resistance (Figure S5). Interestingly, while the two acquired drug-resistant derivatives achieved a similar high degree of resistance to PLB treatment (250 nM PLB GR values: 0.709 vs. 0.778 for the VOA6406_TRA_SR/VOA6406_PLB_SR cell line pair), the acquired PLB-resistant cell line achieved a much lower degree of resistance to TRA treatment than the acquired TRA-resistant cell line (50 nM TRA GR values: 0.434 vs. 0.830, respectively) (Table S6). Furthermore, as observed in the parental cell line, the acquired PLB-resistant cell line continued to respond synergistically to PLB and TRA combination (Figure 4B and Figure S5B). At the signaling level, the development of acquired-PLB resistance resulted in an increase in cyclin E1 expression, which further increased under high-dose PLB (250 nM, 48 h) treatment (Figure 4D). However, no obvious changes in total or phosphorylated MAPK or Rb levels were noted (Figure 4D and Figure S5B).

### 2.5. Combination of PLB and TRA Treatment Exerted Superior Cell Inhibitory Effects than Single-Agent Treatment in a KRAS-Wild-Type/NRAS-Mutant Xenograft Model of LGSOC

**Figure 5:**
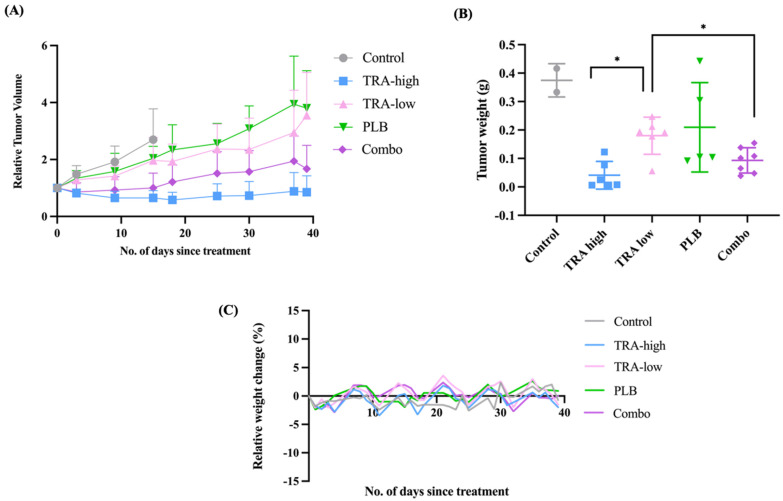
Preclinical efficacy of TRA and PLB drug therapy in a LGSOC xenograft model. (**A**) Mice were implanted with VOA6406 CDX and treated for 39 days with TRA 0.5 mg/kg (TRA-high), TRA 0.05 mg/kg (TRA-low), PLB 20 mg/kg, or combo (TRA-low plus PLB combination). Results are presented as changes in relative tumor volume with error bars, standard deviation (SD). (**B**) Dry weights of the tumors in different groups at study termination. (**C**) Mean percent of weight change in the mice in different groups during the experiment. The mice were treated 5 days on, 2 days off. (*) *p*-value of less than 0.05 was considered statistically significant.

To validate the potential efficacy of the TRA and PLB combination for the treatment of LGSOC, we evaluated these therapies in the cell-derived xenograft (CDX) of VOA6406 (*KRAS*-wt, *NRAS*-mut), with which the synergistic effect of these drugs was demonstrated (Figure 3A). As previously reported by our team and collaborators, VOA6406 CDX was responsive to TRA treatment at 0.5 mg/kg [40]. Therefore, this treatment group was included as comparison. To evaluate the combination effect of TRA and PLB, a 10-fold lower dose of TRA at 0.05 mg/kg and a low dose of PLB at 20 mg/kg were used [27,41]. During the course of this experiment, four out of six CDX mice in the control group (vehicle) developed skin ulcerations from progressive disease and had to be euthanized. Remarkably, none of the CDXs treated with the single agents or the combination ulcerated, presumably due to the activity of the drugs. Single treatment of the CDXs with PLB (20 mg/kg) or TRA (0.05 mg/kg) did not seem to change the trajectories of the tumors (Figure 5A). When the CDXs were treated with the PLB (20 mg/kg) + TRA (0.05 mg/kg) combination, the relative growth of the xenografts was significantly inhibited when compared to the single-agent groups (Figure 5A). The inhibitory effect of the combination therapy is confirmed by the significant reduction in final dry tumor weight as defined before (TRA-low vs. TRA-low + PLB *t*-test = 0.02335) (Figure 5B) [40]. The mice tolerated the single treatments and combination treatment well, demonstrated by the stable weight (Figure 5C). The results showed that the PLB + TRA combination, with 1/10 of the effective TRA dose, was non-inferior to the single high TRA dose, and it was superior in exerting the inhibitory effect on the VOA6406 CDX when compared to the single-agent PLB.

## 3. Discussion

Despite advancements in cancer research, the treatment of rare cancers has often been based on using drug therapies that have been developed to target molecular drivers found in common cancer types. This approach has accelerated the development and approval of therapies for rare cancers by leveraging existing knowledge and data from larger patient populations [42,43]. In LGSOC, molecular profiling revealed marked similarities with hormone receptor–positive breast cancer and MAPK-driven colorectal cancer, which resulted in the clinical evaluation and approval of AHT and MEKi as standard treatments for LGSOC patients [18]. However, MEKi therapy has resulted in modest responses rates (26%) and PFS (median = 13 months), highlighting the urgent need for predictive biomarker research and additional treatment strategies [10]. Similarly, building on the insights from other cancer types, CDK4/6i is now being evaluated alone and in combination (e.g., MEKi, AHT) in several clinical trials involving LGSOC patients [23,26,30]. While early clinical trial results are promising, clinically validated biomarkers for predicting drug efficacy remain a key research challenge [23,26,30].

In our previous work, we explored MEKi efficacy in patient-derived LGSOC models and identified biomarkers of MEKi resistance (including EGFR, PKCα, and NOTCH) and novel putative disease drivers (such as *CDKN2A*/p16 loss) [9,34,35,36,40]. These findings provided rationale for investigating CDK4/6i, alone and in combination with MEKi, as a potential therapeutic opportunity in LGSOC.

In the present study, we found that primary-advanced LGSOC tumors with abnormal p16 expression (loss or block IHC staining) were associated with poorer survival outcomes (19.6%, 29/146, *p* = 0.005). Interestingly, p16 abnormalities were nearly three times more frequent in recurrent tumors (46.2%) compared to matched primary tumors (15.4%), although it is not known whether this is due to natural tumor evolution or an effect of treatment. LGSOC tumors with abnormal p16 expression were more amenable to cell line establishment, suggesting that loss of *CDKN2A*/p16 may confer a growth advantage in vitro. These findings are consistent with previous reports of our own and others in which alterations of *CDKN2A*/p16 were associated with worse survival in LGSOC and other cancers [9,33,44,45,46]. Moreover, recent preclinical research implicates p16 loss as a strong predictive biomarker of CDK4/6i sensitivity in several cancer types [47]. Thus, future studies are needed to validate the prognostic and predictive significance of p16 expression in LGSOC. Of note, 20% of primary-advanced LGSOC tumors expressed high cyclin D1 levels (>50% positive cells by IHC). Estrogen signaling is known to induce cyclin D1 expression, which has been associated with AHT resistance in estrogen receptor–positive breast cancer [48]. As AHT is a standard treatment for primary-advanced LGSOC, this finding should be further investigated as potential biomarker of AHT resistance.

Our preclinical analyses of LGSOC cell lines found that most lines displayed abnormal p16 expression (9/10, absence or block); however, these had notable differences in Rb protein levels (total and phosphorylated Rb). In particular, *KRAS/NF1*-wild-type cell lines exhibited higher Rb phosphorylation and lower E1 levels compared to the *KRAS/NF1*-mutant cell lines. In all cell lines, PLB monotherapy elicited modest antiproliferative effects (n = 6), whereas TRA monotherapy was more effective in the *KRAS/NF1*-mutant cell lines (n = 3) compared to the *KRAS/NF1*-wild-type lines (n = 3). As expected, TRA treatment reduced cyclin D1 levels (n = 2), as cyclin D1 is a known downstream target of MAPK signaling [49]. Notably, cyclin D1 expression was not affected in the cell lines with acquired TRA resistance. TRA and PLB in combination demonstrated synergistic antiproliferative effects only in the *KRAS/NF1*-wild-type cell lines (n = 3). This drug synergy was confirmed in vivo, where low-dose PLB and TRA combination achieved comparable growth inhibition to high-dose TRA monotherapy. Thus, our findings describe the existence of two groups of LGSOC cell lines characterized by their MAPK status and cell cycle pathway activation (Rb phosphorylation).

Next, we further explored molecular determinants of drug response. Functional analyses in two representative LGSOC cell lines (one *KRAS*-mutant and one wild-type) provided additional insight into the potential mechanisms of efficacy of TRA and PLB treatments. Here, we confirmed that *KRAS/NF1*-mutant LGSOC cell lines are more sensitive to TRA (n = 3) [35,36] and that TRA sensitivity was associated with complete inhibition of pRb expression (n = 1). Previous studies have demonstrated that MEKi shows greater growth inhibition in *KRAS* mutants (like *KRAS*-driven lung, colorectal cancers) compared to *RAS*-wild-type cancers [27]. Additionally, preclinical studies in *KRAS*-mutated colorectal cancer and lung adenocarcinoma cell lines showed that TRA efficacy was associated with decreased Rb phosphorylation [49,50,51,52]. Remarkably, we also found that only the *KRAS/NF1*-wild-type LGSOC cell lines responded synergistically to the PLB and TRA combination (n = 3), and that this was linked to the negative effects of PLB on MAPK phosphorylation (n = 1). In support of this observation, studies in other cancer types (breast cancer, colorectal cancer and glioblastoma) have shown that PLB can result in the inhibition of both pRb and pMAPK expression [50,53,54]. Of further importance, studies in breast cancer have also shown that activating mutations of the RAS/MAPK pathway (such as *KRAS* and *NF1*) are associated with resistance to CDK4/6i treatment [28,55,56]. Recently, functional genetic screens performed in melanoma cell lines demonstrated that activated *KRAS* induced resistance to CDK4/6i treatment when administered as monotherapy or in combination with MEKi treatment [57].

In this study, we also examined cyclin E1 expression in relation to the efficacy of MEKi and CDK4/6i treatment. Most primary-advanced LGSOC tumors expressed low levels of cyclin E1 (<25% positive cells) by IHC. However, LGSOC cell lines with *KRAS/NF1* mutation showed higher levels of cyclin E1 expression than the *KRAS/NF1*-wild-type cell lines. Upregulation of cyclin E1 expression was also observed in the *KRAS/NF1*-wild-type cell line after the development of the acquired resistance to PLB treatment. High cyclin E1 expression (resulting from *CCNE1* gene amplification or transcriptional regulation) is known to promote resistance to CDK4/6 inhibition [58,59,60,61]. These findings provide an additional explanation for the lack of drug synergy observed in the *KRAS/NF1*-mutant cell lines and suggest that cyclin E1 expression could serve as a predictive biomarker of CDK4/6i efficacy in LGSOC. In support of our findings, a recent clinical trial evaluating PLB in recurrent ovarian cancer patients demonstrated that PLB alone had modest activity in unselected patients [62]. Exploratory tumor profiling identified potential predictors of sensitivity (*CDKN2A* deletion) and resistance (*CCNE1* amplification or *RB1* deletion), warranting further investigation.

The limitations of this study should be acknowledged and are primarily related to the following factors: (1) the limited number of available LGSOC preclinical models which represent tumors treated with different therapies; (2) LGSOC cell line models lose estrogen receptor (ER) expression in vitro [63]; and (3) the lack of models retaining p16 expression, as they are difficult to establish in vitro. Despite frequent ER expression in recurrent LGSOC (58–96% expression; >1% positive cells), clinical RR to AHT are dismal (2–13% RR) [4,64]. Taken together, these limitations support the use of the current available models for evaluating therapies in the relapsed disease setting. However, caution should be exercised when generalizing our findings to patients treated with PLB in the primary setting as these patients more frequently have functional ER.

The two most promising strategies for treating recurrent LGSOC are currently MAPK pathway inhibitors and CDK4/6i. The identification of predictive biomarkers to guide treatment selection in LGSOC is paramount and challenging. Combination therapy with MEKi and CDK4/6i has been proposed to overcome the CDK4/6i resistance linked to MAPK pathway alterations [65]. Thus, the comboMATCH study was designed to include patients with *KRAS*-mutated LGSOC [30]. Remarkably, we only observed MEKi and CDK4/6i combination synergy in the *KRAS/NF1*-wild-type models, suggesting that this group of patients is more likely to benefit from this treatment.

Recently the FDA approved a novel therapy for *KRAS*-mutated relapsed LGSOC. The RAMP 201 trial reported that the combination of avutometinib (MEK/RAF clamp) plus defactinib (FAK inhibitor) resulted in a 44% response rate in the *KRAS*-mutated tumors, however the response rate in the *KRAS*-wild-type patients was only 17% [66]. These results suggest that *KRAS* mutations are a predictive biomarker for this therapy. Within the KRAS-wild-type LGSOC population there are several lines of evidence that suggest CDK4/6 inhibitors should be evaluated: (1) we observed higher cyclin E1 levels in the *KRAS/NF1*-mutated tumors; (2) according to the literature, RAS activation can mediate resistance to CDK4/6i [59]; (3) we observed synergistic activity with the addition of a CDK4/6i to MEKi in the *KRAS*-wild-type models; and (4) response rates to avutometinib/defactinib combination are low in *KRAS*-wild-type patients [66]. Additionally, CDK4/6i has also shown promise in relapsed LGSOC when combined with AHT. A recent clinical trial, GOG 3026, evaluated letrozole (AHT) in combination with ribociclib (CDK4/6i) which resulted in a 30% RR among 48 patients with a median PFS of 19.1 months [23]. Another trial reported a higher response rate of 47% when abemaciclib (CDK4/6i) was combined with fulvestrant (AHT) in a small neoadjuvant study [26]. The use of biomarkers to select patients for CDK4/6i therapy, in combination with MEKi or AHT, is a critical research challenge. Clinical trials using CDK4/6i must include translational biomarker studies to help identify those LGSOC patients who may benefit from CDK4/6 treatment.

In summary, our study provides preclinical evidence of the efficacy of TRA and PLB therapies for treating LGSOC. This work has helped elucidate potential biomarkers for treatment selection and future study. MEKi monotherapy remains most active in *KRAS*-mutated LGSOC, reinforcing MAPK pathway activation *(KRAS/NF1)* as a predictive biomarker of MEK-directed therapies. Our data also suggests that tumors lacking these mutations may derive benefit CDK4/6i therapy. Thus, identification of MAPK pathway activating mutations (*KRAS/NF1*) and cell cycle pathway proteins (p16, Rb, cyclin D1, cyclin E1) may provide a rationale framework to guide the use of MEKi- and CDK4/6i-based therapies in LGSOC. The confirmation of our findings, including specific biomarker validation, could help inform the design and interpretation of current and future clinical trials, resulting in better outcomes for patients with LGSOC.

## 4. Methods and Materials

### 4.1. The Patients, Tumor Samples, and Clinical Information

Advanced or recurrent LGSOC samples (tumors and ascites) were obtained from the Gynecology Tumor Bank and the Department of Pathology of The University of British Columbia (British Columbia), and the Department of Pathology and Laboratory Medicine of the University of Calgary (Alberta) [13]. Tumor bank protocols, cell line derivation, and the research relating to this study was conducted according to institutional human ethics review board approvals by Health and Research Ethics Board Alberta (HREBA.CC-16-0161, HREBA.CC16.0159, HREBA.CC-16.0371, HREBA.CC-21-0362) and the University of British Columbia Ethics Board (H19-02823, H22-00544, H17-01863, H18-00280). Clinical information was extracted retrospectively from patient medical records. The pathology diagnosis for all cases in this study were reported on or reviewed by a gynecological pathologist.

### 4.2. Establishment and Maintenance of Patient-Derived LGSOC Cell Lines

LGSOC patient-derived cell lines were previously established in-house through a continuous in vitro culture of primary patient material (tumor tissue or ascites) obtained through the Gynecology Tumor Bank or the London Translational Ovarian Cancer Research Program (for cell line iOvCa241). Three new LGSOC cell lines (VOA10841, VOA13738 and VOA14202) were established and maintained using our previously published protocols without the use of immortalization methods [35,36]. For quality control purposes, all cell lines were confirmed to be mycoplasma-free using the MycoProbe Mycoplasma Detection Kit (R&D Systems, Minneapolis, MN, USA) before freezing. Cell line authentication was performed using Microsatellite Analysis of Short Tandem Repeats (STRs) of 10 markers/loci by Genewiz Inc. (South Plainfield, NJ, USA).

### 4.3. Immunohistochemistry

Five ovarian cancer TMAs (2 from Calgary, 2 from Vancouver and 1 from COEUR) containing 186 LGSOC and duplicate 0.6 mm tissue cores for each tumor were used in this study. With the exception of the COEUR TMA, for which only p16 data was obtained, for each TMA 4 sections (4 microns in thickness) were stained against the following markers: p16, Cyclin D1, Cyclin E1 and Rb. Antibodies and dilutions are detailed in Table S7A. IHC was conducted using the DAKO Omnis platform according to the standard procedures. Centralized tumor IHC staining and scoring was performed by a single pathologist (MK). The highest score for a given case was used for analysis. As previously published, classification of p16 staining was based on a 3-tier system (PS2+ scoring): complete absence, normal (patchy heterogenous staining), and block staining (strong, diffuse nuclear and cytoplasmic signal) [13]. Both absent or overexpressed (block-like) p16 IHC patterns are considered abnormal [33]. Classification of Rb staining was as follows: loss, retained, sub-clonal loss, or equivocal. Staining of Cyclin D1 and Cyclin E1 were based on the percent of positive tumor cell nuclei and arbitrary defined as follows: low, <25% positive cells; moderate, 25–50% positive cells; and high, >50% positive cells. Finally, for 26 primary LGSOC tumors, p16 IHC staining was conducted in their corresponding recurrent tumor using independent tissue sections.

### 4.4. Statistical Tests

Demographics and baseline characteristics were summarized using descriptive statistics (total number or N, median, and range) for continuous variables and N (%) for discrete variables. Associations of p16 IHC expression with demographic and clinical variables were examined using the Chi-Square test. Descriptive analysis between p16 staining values and other clinical and molecular variables was assessed using Pearson correlation coefficients. Clinical variables included: age (continuous), tumor type (primary, recurrent), FIGO stage (I–II, III–IV, unknown), residual disease (absent, optimal < 1 cm, suboptimal >1 cm, unknown), tumor site of origin (ovarian, peritoneal, unknown) and treatment type (chemotherapy naïve, post neoadjuvant chemotherapy, post other treatments, unknown). Primary end point for overall survival (OS) analysis was death by any cause (date of diagnosis to date of death by any cause/date of last follow-up) and was reported in months. Survival outcomes were determined through clinical chart review. Kaplan–Meier survival curves and corresponding log-rank tests were generated to visually assess associations of p16 expression with survival. Cox proportional hazards model was used for multivariable assessment of hazard ratios. Drug treatment responses were compared using *t*-tests and a *p*-value of less than 0.05 was considered statistically significant.

### 4.5. Sequencing of Newly Established Patient-Derived LGSOC Cell Lines

For the previously developed lines, sequencing was performed as described by Shrestha et al. [34]. For the newly developed lines (VOA10841, VOA13738, VOA14202), DNA was extracted from patient tumor, cell line and matching buffy coats using an All Prep DNA/RNA Mini kit (Cat. No. 80204, Qiagen, Toronto, ON, Canada) and DNeasy Blood and Tissue Kit (Cat No. 69504, Qiagen, Toronto, ON, Canada), respectively, according to protocol instructions, and quantified using a NanoDrop 2000TM UV–Vis instrument (Thermo Scientific, Burlington, ON, Canada).

### 4.6. Development of LGSOC Cell Lines with Acquired Resistance to Trametinib (TRA) and Palbociclib (PLB)

The iOvCa241_TRA_R cell line was generated in Dr. Mark Carey’s laboratory from a patient-derived LGSOC cell line (iOvCa241; *KRAS*-mutant) established in Dr. Gabriel DiMattia’s laboratory [36]. The VOA6406_TRA_SR cell line was generated by our research collaborators at the Netherlands Cancer Institute (clone R18) from a patient-derived LGSOC cell line (VOA6406; *KRAS*-wild-type, *NRAS*-mutant) established in our laboratory [40]. The VOA6406_PLB_SR cell line was generated in-house from the VOA6404 cell line. All drug-resistant cell lines were obtained by continuous culture in M199:MCDB105 media with 10% FBS under drug selection: TRA (GSK1120212; Cedarlane; Sellekchem, Cat. No. S2673) or PLB (Cedarlane; Selleckchem, Cat. No. S1116). As in the protocol outlined by Coley et al., increasing amounts of drug (TRA or PLB) were used until reaching the following final drug tolerances: 20 nM TRA in the iOvCa241_TRA_R and VOA6406_TRA_SR cell lines, and 1.0 µM PLB in the VOA6406_PLB_SR cell line [67]. Confirmation of acquired TRA and PLB resistance was determined by live-cell imaging, cell counting, and cell viability assays.

### 4.7. Cell Counting Assays

In the six LGSOC cell line models tested, PLB treatment induced cell morphology changes that resemble a senescent-like state characterized by cellular flattening and enlargement that was visible under the microscope. As previously reported in the literature, these changes are known to interfere with commonly used laboratory read out measurements of drug efficacy (such as those obtained from IC50 assays) [47,68,69]. To overcome this limitation, and as described above for TRA evaluation, we conducted drug efficacy determinations of PLB, alone and in combination with TRA, using cell counting and GR corrections. To do so, LGSOC cell lines were seeded in 48-well plates to achieve 15–25% initial confluence 24 h post-seeding (6000–28,000 cells/well depending on the cell line). Initial cell confluence was measured by live-cell imaging using Incucyte™ ZOOM 2016B (Sartorius–Essen Biosciences, Ann Arbor, MI, USA). Once the initial confluence was achieved, cells were treated once with DMSO (control, 0.5%), single-agent TRA, single PLB and five different combination doses of PLB + TRA for a duration of 90 h. PLB (PD-0332991 HCl) and TRA (GSK1120212) were purchased from Selleckchem. The maximum PLB and TRA concentrations used in this study were chosen to closely reflect the calculated steady-state patient serum levels or maximum tolerated doses observed in clinical trials (259 nM for PLB and 35.1 nM for TRA) [70,71,72]. TRA doses ranged from 5.5 nM to 50 nM, PLB doses ranged from 62.5 nM to 250 nM. Combination doses were 125 nM PLB + 5.5 nM TRA, 125 nM PLB + 16.6 nM TRA, 250 nM PLB + 5.5 nM TRA, 250 nM PLB + 16.6 nM TRA, and 250 nM PLB + 50 nM TRA. Within each experiment, each drug condition had three technical replicates and three biological replicates were run for each cell line to confirm the reproducibility of findings. At the end of the experiment individual wells were trypsinized and counted using a BioRad TC-10 automated cell counter.

### 4.8. Growth Rate Calculations and Drug Synergy Analysis

Cell count measurements were conducted at the beginning and at the end of each cell counting experiment as described in Methods 4.7 (90 h post-treatment). To control for variability in cell doubling times between LGSOC cell lines, GR adjustments were calculated for each cell line and drug treatment condition using raw cell counting data and the growth rate calculator developed by Hafner et al. 2016 (grcalculator.org) [73]. A GR value between 1 and 0 signifies a partially cytostatic response, where 0 indicates a complete cytostatic response. A negative GR value signifies a cytotoxic effect and a GR value of >1 signifies that the drug treatment promotes cell growth. Data was presented as mean ± standard error of the mean (SEM) and analyses were performed in GraphPad Prism (version 10) using a multiple paired *t*-test; statistical significance was defined as *p* < 0.05. Using GR values of single and combination drug doses, drug synergy was calculated with the CompuSyn software [74]. A CI < 1 indicates synergistic drug combination effects, whereas an additive drug effect is reported as a CI value equal to 1 and a CI > 1 indicates an antagonistic effect.

### 4.9. CDK Drug Screening

CDK drug screening was performed as previously described at the Victorian Centre for Functional Genomics (Peter MacCallum Cancer Institute, Australia) [37]. Briefly, cells were seeded in 384-well plates, left to adhere overnight, and treated with CDK compounds (Compounds Australia, Griffith University, Australia) provided in assay ready (0.1, 1 and 10µM) dry format. Following 72 h incubation, cells were stained with DAPI with cell imaging performed on a CellInsight CX7 LED high content imager. All cell seeding, drug plate rehydration and fixing/staining liquid handling steps were performed using a robotic BioTek 406 liquid handling platform (BioTek, Agilent, Santa Clara, CA, United States). Images were analyzed for cell counts using the open-source modular image analysis software CellProfiler (version 4.1.3) with nuclear identification and segmentation settings customized for each cell line. Viability was assessed through robust Z-score  =  (sample value-median of all sample values)/median absolute deviation of all sample values.

### 4.10. Western Blot Analysis

WB were performed as previously described [35,36]. Protein cell lysates were extracted from sub-confluent cell culture plates. Treated cell lysates were exposed to 48 h drug treatment before extraction. Antibodies and dilutions are detailed in Table S7B. WB were imaged using Immobilon HRP reagent (Cat. No. WBKLS0500, Millipore, Etobicoke, ON, Canada) and developed by autoradiograph. 

### 4.11. LGSOC Xenograft Model and In Vivo Experiment

The VOA6406 xenograft model was developed from serial transplantation of the xenografts in immunocompromised mice from the initial cell-derived tumor [40]. Actively growing VOA6406 tumors were harvested and cut into pieces of 2 × 2 × 2 mm in dimension and implanted subcutaneously in the cohort of experimental mice (NOD.Cg-Rag1tm1Mom Il2rgtm1Wjl/SzJ). Once the tumors reached an average size of 100 to 150 mm^3^, the mice were assigned into 5 treatment groups with 6–7 mice/group: vehicle control, TRA (low, 0.05 mg/kg; high, 0.5 mg/kg), PLB (20mg/kg), and TRA (0.05 mg/kg) + PLB (20 mg/kg). Drug combinations were formulated in DMSO: Kolliphor EL (Sigma 27963): saline solution in a 1:1:8 ratio. The experimental mice were treated 5 days a week by intraperitoneal injection with weekend breaks. Tumor growth was quantified once a week by caliper measurements according to the approximation formula: tumor volume = (width^2^ × length)/2. Relative tumor growth was normalized to the tumor volume of each tumor at the start of treatment. At the experimental endpoint, all tumors were harvested. The “dry” weight of each tumor was measured with the exclusion of the cystic fluid. Statistical significance for tumor weight differences was determined using a two-tailed *t*-test. The animal studies were carried out at Animal Resources Centre at BC Cancer, Vancouver, according to animal ethics certificates A22-0156 and A22-0204 approved by the Animal Care Committee at the University of British Columbia.

## Figures and Tables

**Table 1:** Immunohistochemistry results of cell cycle proteins in primary-advanced (III–IV) LGSOC tumors (n = 146). Staining of p16 was performed in 5 independent TMAs (n = 146 tumors). (*) Staining of cyclin D1, cyclin E1, and Rb1 was performed in 4 TMAs (n = 90 tumors); not done in the COEUR TMA.

Protein Name	IHC Expression	Number of Tumors, *n* (%)
p16	Normal	117/146 (80.1%)
	Abnormal	29/146 (19.9%)
	Absence	23/146 (15.8%)
	Block	6/146 (4.1%)
	Not done *	0
	Uninterpretable	9
	Total	155
Cyclin D1	Low	44/87 (50.6%)
	Moderate	24/87 (27.6%)
	High	19/87 (21.8%)
	Not done *	56
	Uninterpretable	12
	Total	155
Cyclin E1	Low	81/82 (98.8%)
	Moderate	0/82 (0%)
	High	1/82 (1.1%)
	Not done *	56
	Uninterpretable	17
	Total	155
Rb1	Normal	85/85 (100%)
	Abnormal	0/85 (0%)
	Loss	0/85 (0%)
	Sub-clonal loss	0/85 (0%)
	Not done *	56
	Uninterpretable	14
	Total	155

**Table 2:** Immunohistochemistry (IHC) results of p16 in matched primary and recurrent LGSOC tumors (n = 26 patients). Among these cases, p16 IHC aberrations were detected in over 15% of primary and 46% of recurrent tumors representing a 3-fold increase in their frequency. Acquisition of p16 IHC aberrations at the time of recurrence was detected in 30% of cases. Data on primary cases was obtained from TMA cores and data on recurrent cases was obtained from whole tumor sections.

		p16 IHC PS2+ Scores		
LGSOC ID	Tumor Stage	Primary (n = 26)	Recurrent (n = 26)	Score Change (Yes/No)
LGSCv2_1	3	Normal	Normal	No
LGSCv2_2	1	Normal	Absence	Yes
LGSCv2_3	3	Normal	Absence	Yes
LGSCv2_6	3	Normal	Normal	No
LGSCv2_14	3	Normal	Absence	Yes
LGSCv2_22	3	Normal	Normal	No
LGSCv2_28	3	Normal	Normal	No
LGSCv3_3	3	Normal	Normal	No
LGSCv3_12	4	Normal	Absence	Yes
LGSCv3_14	3	Normal	Normal	No
LGSCv3_16	3	Absence	Absence	No
LGSCv3_21	4	Normal	Normal	No
LGSCv3_25	3	Normal	Normal	No
LGSCv3_27	3	Absence	Absence	No
LGSCv3_28	3	Normal	Absence	Yes
LGSCv3_30	2	Block	Block	No
LGSCv3_33	3	Normal	Normal	No
LGSCv3_34	3	Normal	Normal	No
LGSCv3_35	3	Normal	Normal	No
LGSCv3_36	3	Normal	Absence	Yes
LGSCv3_38	3	Normal	Block	Yes
LGSCv3_43	3	Normal	Normal	No
LGSCv3_45	3	Normal	Normal	No
LGSCv3_46	3	Normal	Normal	No
LGSCv3_47	3	Absence	Absence	No
LGSCv3_48	3	Normal	Absence	Yes
Normal, n (%)		22/26 (84.6%)	14/26 (53.8%)	-
Abnormal, absence, n (%)		3/26 (11.5%)	10/26 (38.5%)	-
Abnormal, block, n (%)		1/26 (3.8%)	2/26 (7.7%)	-
Abnormal total (absence + block), n (%)		4/26 (15.4%)	12/26 (46.2%)	-
Acquired p16 abnormality (absent or block), n (%)		-	-	8/26 (30.8%)

**Table 3:** LGSOC patient demographics and their associations with p16 tumor expression. P16 data was obtained from primary and recurrent tumors. Demographics data belongs to primary cases only. (~) Primary LGSOC cases from tissue microarrays (TMAs), all stages. (**) Primary-matched recurrent cases stained outside the TMAs (whole tumor sections). (*) Pearson Chi-Square *p*-value significance lower than 0.05, p16 normal vs. total abnormal values. (^) Tumor of origin not reported for the COEUR dataset. N (n) = number of independent cases.

LGSOC Patient’s Information		N	%	1-Normal, n (%)	2-Abnormal, Absent, n (%)	3-Abnormal, Block, n (%)	2- and 3-Abnormal, Total, n (%)	*p*-Value
Age (years)	Mean	54	-	-	-	-	-	-
	Standard deviation	13	-	-	-	-	-	-
	Range	21–84	-	-	-	-	-	-
	**Total**	186	-	-	-	-	-	-
Tumor type	Primary ~	177	87.2	141 (79.7%)	27 (15.3%)	9 (5.1%)	36 (20.3%)	<0.001 *
	Recurrent **	26	12.8	14 (53.8%)	10 (38.5%)	2 (7.7%)	12 (46.2%)	
	Missing data	9	-	-	-	-	-	
	**Total (evaluable cases)**	203	100.0	155 (76.4%)	37 (18.2%)	11 (5.4%)	48 (23.6%)	
Stage	I–II	27	15.6	21 (77.8%)	3 (11.1%)	3 (11.1%)	6 (22.2%)	0.779
	III–IV	146	84.4	117 (80.2%)	23 (15.8%)	6 (4.1%)	29 (19.9%)	
	Missing data	13	-	-	-	-	-	
	**Total (evaluable cases)**	173	100.0	138 (79.8%)	26 (15.0%)	9 (5.2%)	35 (20.2%)	
Residual disease	Absent	41	27.3	34 (82.9%)	5 (12.2%)	2 (4.9%)	7 (17.1%)	0.252
	Optimal < 1cm	64	42.7	49 (75.4%)	12 (18.8%)	3 (4.6%)	15 (23.4%)	
	Suboptimal > 1 cm	45	30.0	40 (88.9%)	4 (8.9%)	1 (2.2%)	5 (11.1%)	
	Missing data	36	-	-	-	-	-	
	**Total (evaluable cases)**	150	100.0	123 (82%)	21 (14%)	6 (4%)	27 (18%)	
Tumor origin	Ovarian	79	76.0	65 (82.3%)	12 (15.2%)	2 (2.5%)	14 (17.7%)	0.265
	Peritoneal	25	24.0	18 (72.0%)	6 (24%)	1 (4%)	7 (28.0%)	
	Missing data ^	82	-	-	-	-	-	
	**Total (evaluable cases)**	104	100.0	87 (83.7%)	18 (17.3%)	3 (2.9%)	21 (20.2%)	
Treatment type	Chemotherapy naïve	145	81.9	115 (79.3%)	22 (15.2%)	8 (5.5%)	30 (20.7%)	0.121
	Post neoadjuvant chemotherapy	30	16.9	25 (83.3%)	4 (13.3%)	1 (3.3%)	5 (16.6%)	
	Post other treatments	2	1.1	1 (50.0%)	1 (50.0%)	0 (0%)	1 (50.0%)	
	Missing data	9	-	-	-	-	-	
	**Total (evaluable cases)**	177	100.0	141 (79.7%)	27 (15.3%)	9 (5.0%)	36 (20.3%)	

## Data Availability

The whole-exome and whole-transcriptome sequencing data from this study are available in the European Genome-phenome Archive, under the following link: https://dataview.ncbi.nlm.nih.gov/object/PRJNA1372646?reviewer=v21b778fh8fgssp6kq566u73b1. (2 February 2026). The public accession number is pending from EGA. FASTQ files from whole-exome sequencing of newly established patient-derived LGSOC cell lines are deposited in the NCBI Sequence Read Archive under BioProject accession PRJNA1372646. Corresponding data for existing cell lines are available from the European Genome-phenome Archive under dataset accession EGAD00001006440. High-throughput drug screening data can be found in the following link DOI: 10.1038/s41597-024-03869-x [37].

## Supplementary Materials

The following supporting information can be downloaded at: https://www.mdpi.com/article/10.3390/ijms27041774/s1.

## Abbreviations

The following abbreviations are used in this manuscript:

AHT	Anti-hormone therapy
BCL2i	BCL-2 inhibitors
CDK46i	Cyclin-dependent kinase 4/6 inhibitors
CDX	Cell-derived mice xenograft
CI	Combination index
FAKi	Focal adhesion kinase inhibitors
GR	Growth rate
HGSOC	High-grade serous ovarian cancer
IHC	Immunohistochemistry
LGSOC	Low-grade serous ovarian cancer
MAPK	Mitogen-activated protein kinase
MEKi	MEK inhibitors
OS	Overall survival
PFS	Progression-free survival
PLB	Palbociclib
Rb	Retinoblastoma
RECIST	Response Evaluation Criteria in Solid Tumors
RR	Response rates
SD	Standard deviation
SEM	Standard error of the mean
TMA	Tissue microarray
TRA	Trametinib
TRA_R	TRA-resistant
TRA_SR	TRA-super resistant
WB	Western blot
