# Supplementary material for: Differential Preclinical Efficacy of Combined CDK4/6 and MEK Inhibition in Low-Grade Serous Ovarian Carcinoma Based on KRAS/NF1 Mutational Status
Source: Int J Mol Sci. 2026 Feb 12;27(4):1774. doi: 10.3390/ijms27041774 (PMC12940316; doi:10.3390/ijms27041774)
Supplement: Supplementary file 1 [file ijms-27-01774-s001.zip › Figures Suppl_Carey MS_2025_Revised_20260129.pptx]

## Slide 1
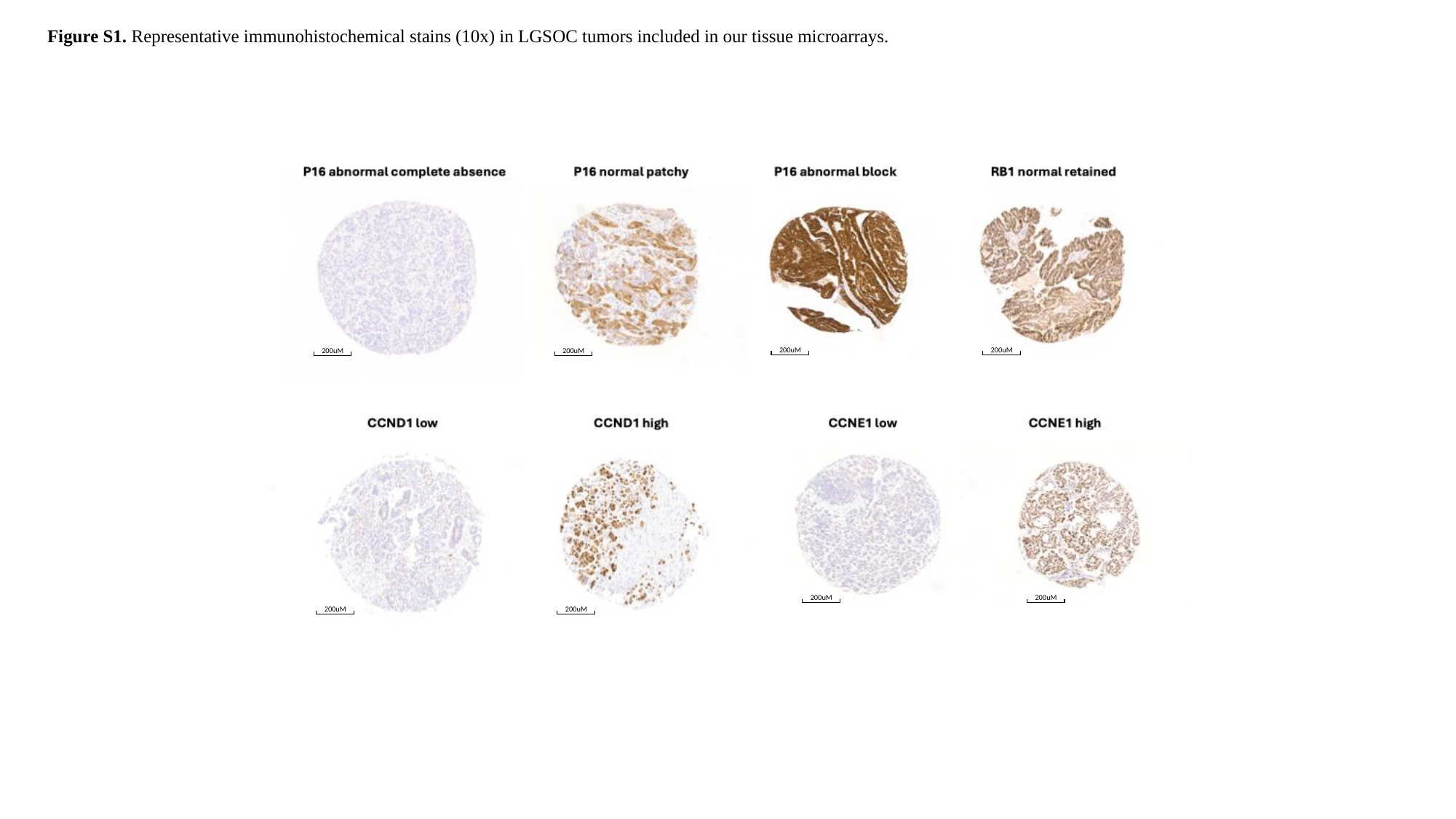

Figure S1. Representative immunohistochemical stains (10x) in LGSOC tumors included in our tissue microarrays.
200uM
200uM
200uM
200uM
200uM
200uM
200uM
200uM

## Slide 2
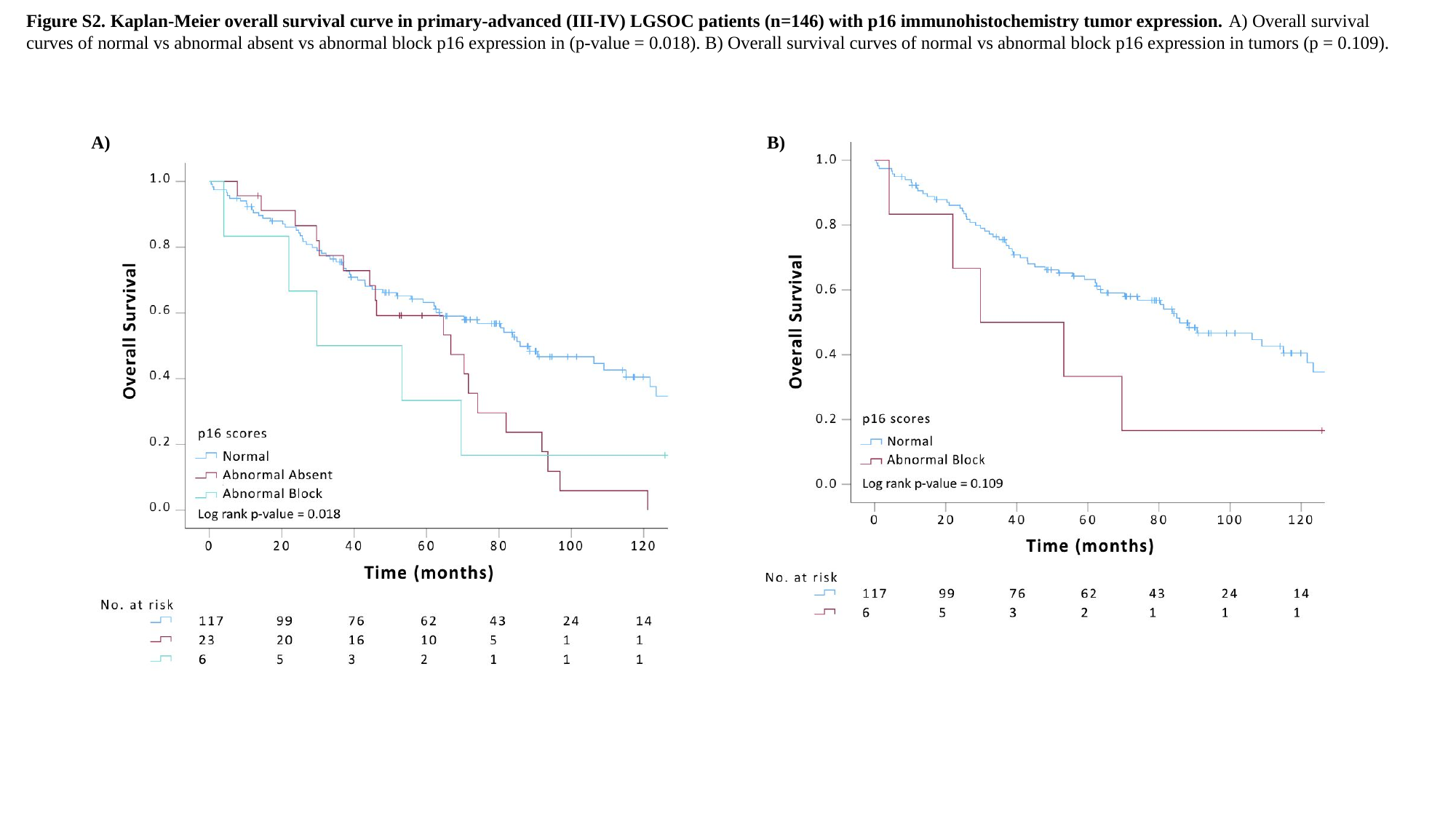

Figure S2. Kaplan-Meier overall survival curve in primary-advanced (III-IV) LGSOC patients (n=146) with p16 immunohistochemistry tumor expression. A) Overall survival curves of normal vs abnormal absent vs abnormal block p16 expression in (p-value = 0.018). B) Overall survival curves of normal vs abnormal block p16 expression in tumors (p = 0.109).
A)
B)

## Slide 3
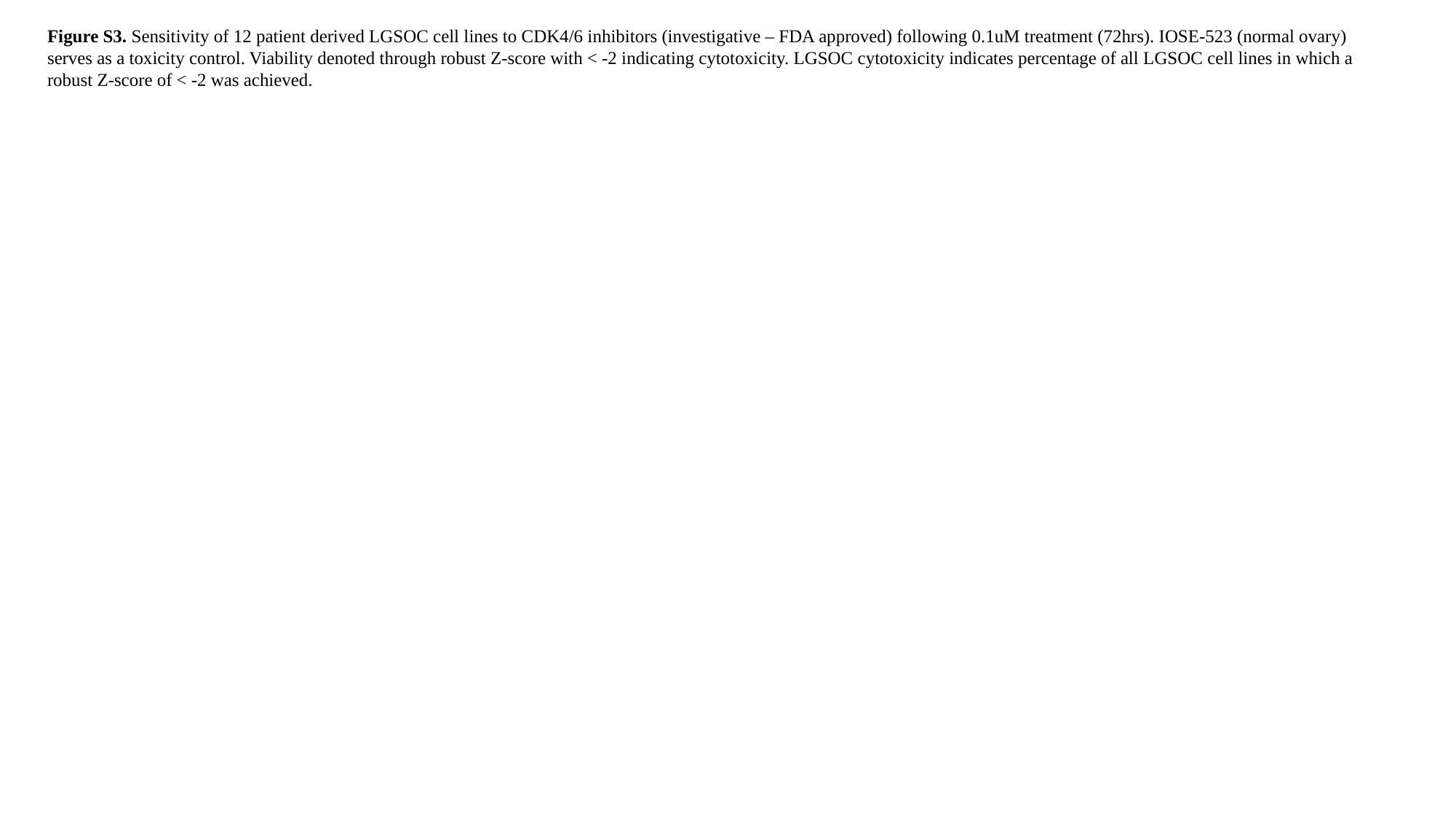

Figure S3. Sensitivity of 12 patient derived LGSOC cell lines to CDK4/6 inhibitors (investigative – FDA approved) following 0.1uM treatment (72hrs). IOSE-523 (normal ovary) serves as a toxicity control. Viability denoted through robust Z-score with < -2 indicating cytotoxicity. LGSOC cytotoxicity indicates percentage of all LGSOC cell lines in which a robust Z-score of < -2 was achieved.

## Slide 4
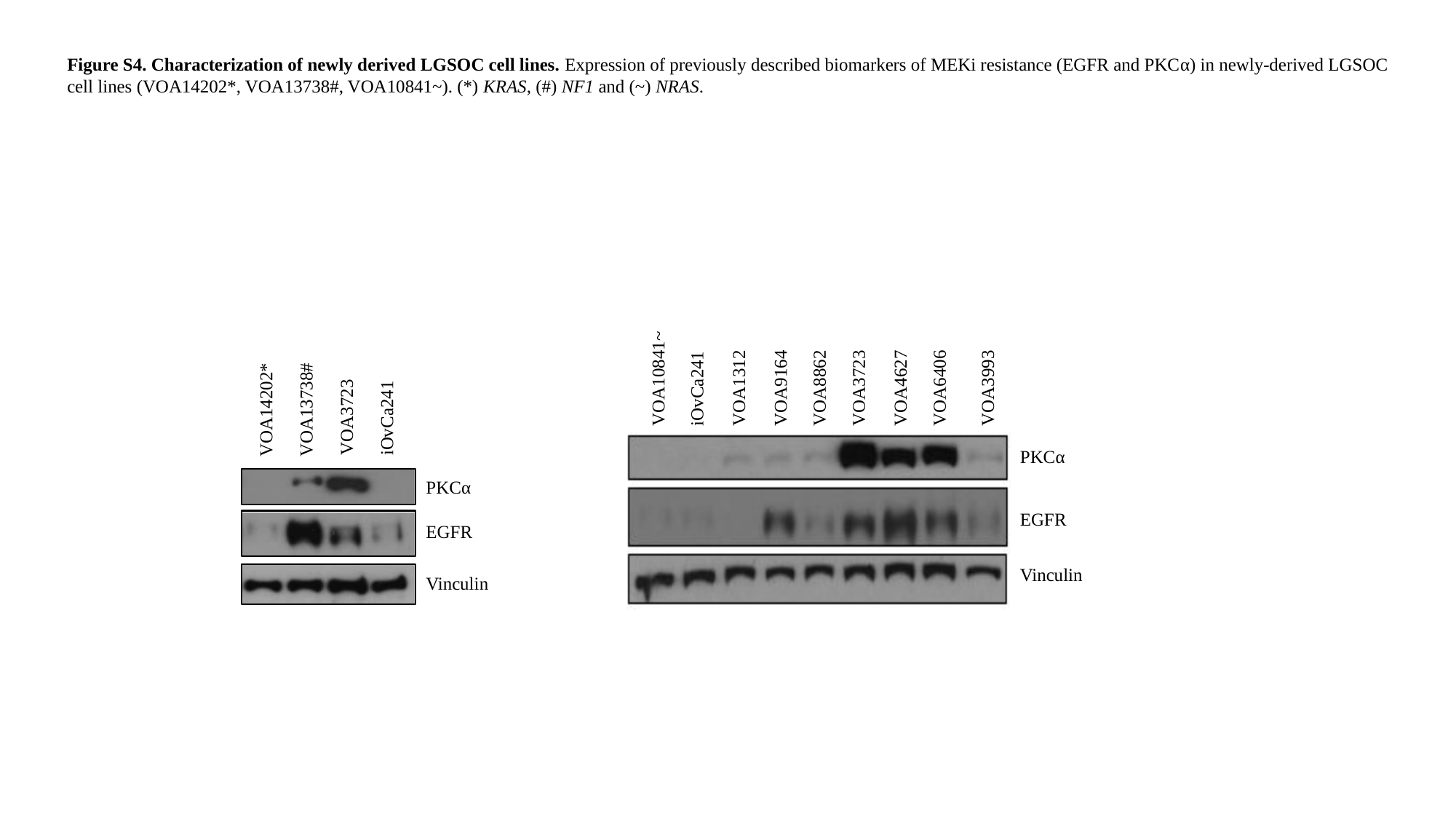

Figure S4. Characterization of newly derived LGSOC cell lines. Expression of previously described biomarkers of MEKi resistance (EGFR and PKCα) in newly-derived LGSOC cell lines (VOA14202*, VOA13738#, VOA10841~). (*) KRAS, (#) NF1 and (~) NRAS.
iOvCa241
VOA3723
VOA14202*
VOA13738#
PKCα
EGFR
Vinculin
VOA1312
VOA10841~
VOA4627
VOA6406
VOA3993
iOvCa241
VOA3723
VOA9164
VOA8862
VOA10841**
iOvCa241**
PKCα
EGFR
Vinculin

## Slide 5
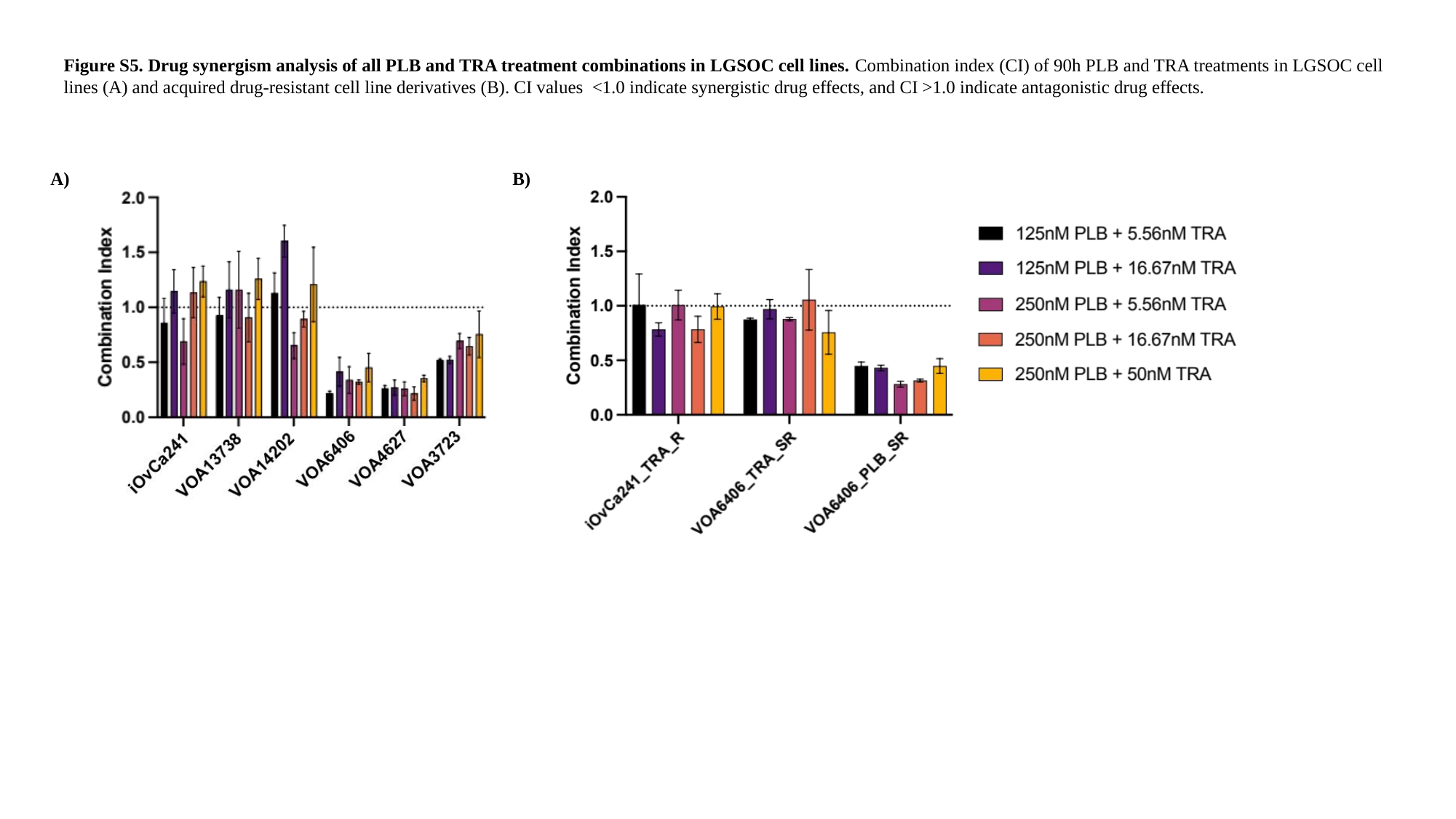

Figure S5. Drug synergism analysis of all PLB and TRA treatment combinations in LGSOC cell lines. Combination index (CI) of 90h PLB and TRA treatments in LGSOC cell lines (A) and acquired drug-resistant cell line derivatives (B). CI values <1.0 indicate synergistic drug effects, and CI >1.0 indicate antagonistic drug effects.
A)
B)

## Slide 6
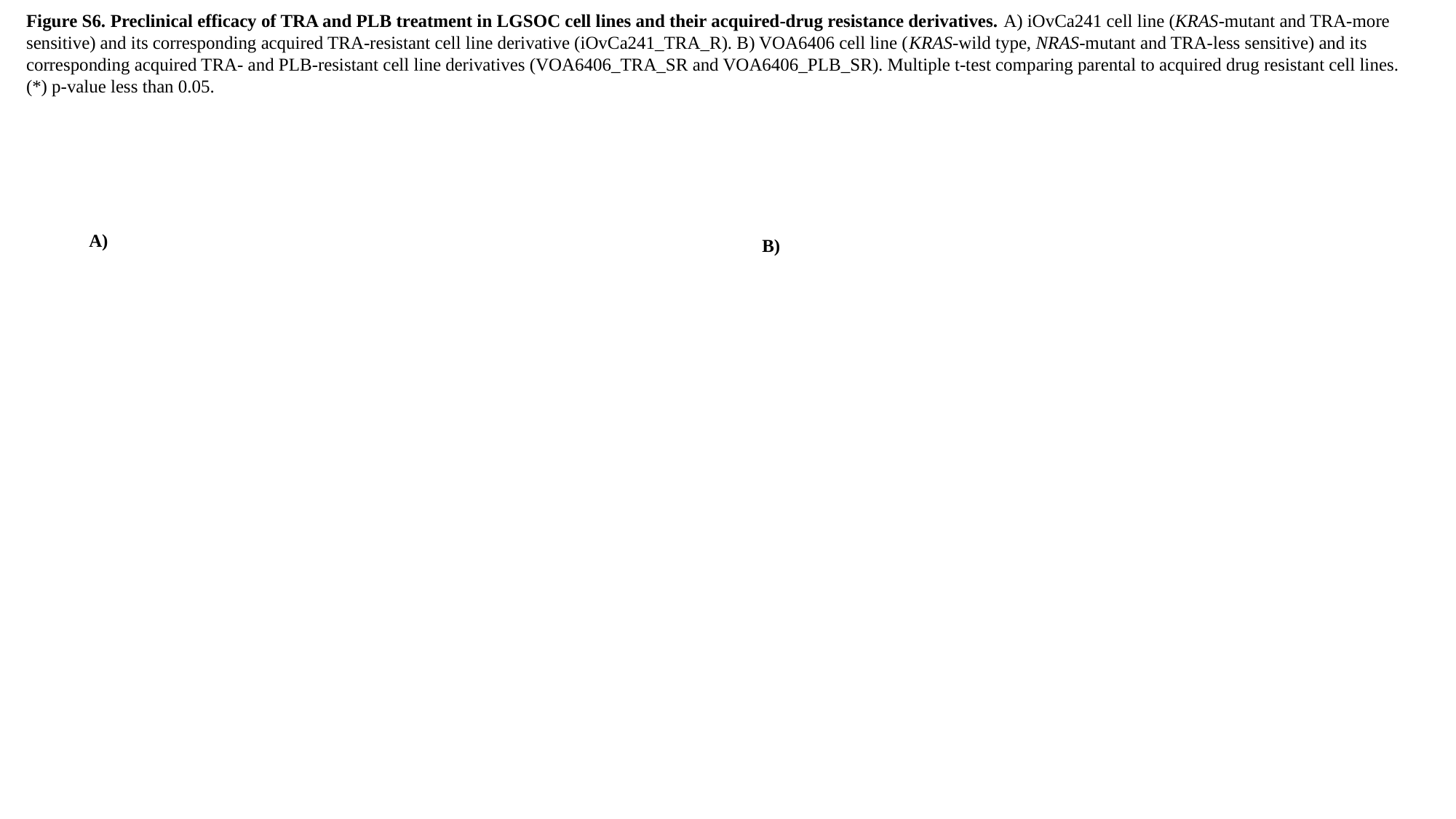

Figure S6. Preclinical efficacy of TRA and PLB treatment in LGSOC cell lines and their acquired-drug resistance derivatives. A) iOvCa241 cell line (KRAS-mutant and TRA-more sensitive) and its corresponding acquired TRA-resistant cell line derivative (iOvCa241_TRA_R). B) VOA6406 cell line (KRAS-wild type, NRAS-mutant and TRA-less sensitive) and its corresponding acquired TRA- and PLB-resistant cell line derivatives (VOA6406_TRA_SR and VOA6406_PLB_SR). Multiple t-test comparing parental to acquired drug resistant cell lines. (*) p-value less than 0.05.
A)
B)
